# Vascular Complications following Isolated Limb Perfusion for Local Recurrence of Extremity Melanoma: A Case Report and Literature Review

**DOI:** 10.1155/2011/204148

**Published:** 2011-07-12

**Authors:** M. Trezzi, A. Parolari, C. Loardi, F. Alamanni

**Affiliations:** ^1^Department of Cardiovascular Surgery, Centro Cardiologico Monzino IRCCS, University of Milan, Via Parea 4, 20138 Milan, Italy; ^2^Unit for Clinical Research in Atherothrombosis, Centro Cardiologico Monzino IRCCS, University of Milan, Via Parea 4, 20138 Milan, Italy

## Abstract

*Introduction*. To evaluate the role of hyperthermic isolated limb perfusion (HILP) in arterial thrombosis following melanoma-soft tissue sarcoma chemotherapy. *Report*. Here is presented one case of iliac-common femoral artery subacute thrombosis and a review of the appropriate literature performed using a MEDLINE search. Acute/subacute arterial occlusion is one of the most feared vascular complications of HILP, located nearly always in the external iliac-femoral artery axis, being those vessels cannulated for perfusion. *Conclusions*. The small number of reported cases indicates either the rarity of this complication or unawareness of its existence. The true incidence of this complication is probably underreported.

## 1. Article

We here present a case of arterial thrombosis following hyperthermic isolated limb perfusion (HILP), a surgical procedure involving an open surgical cannulation of iliac or femoral vessels that is commonly employed in the management of some forms of malignant tumors as extremity sarcoma or melanoma. In particular, HILP is indicated in those patients with melanoma who did not have systemic disease and who may therefore survive for months or years with bulky and unsightly limb metastases that might cause pain and discomfort if HILP is not undertaken [1]. During HILP, high temperature chemotherapeutic agents are recirculated within an extremity and reach tissue concentrations that are 20–30 times higher than during systemic chemotherapy, thereby avoiding any systemic side-effects. Little attention, however, has been paid to vascular complications, which may jeopardize the success of this procedure [2]. 

## 2. Case Description

**Figure 1:**
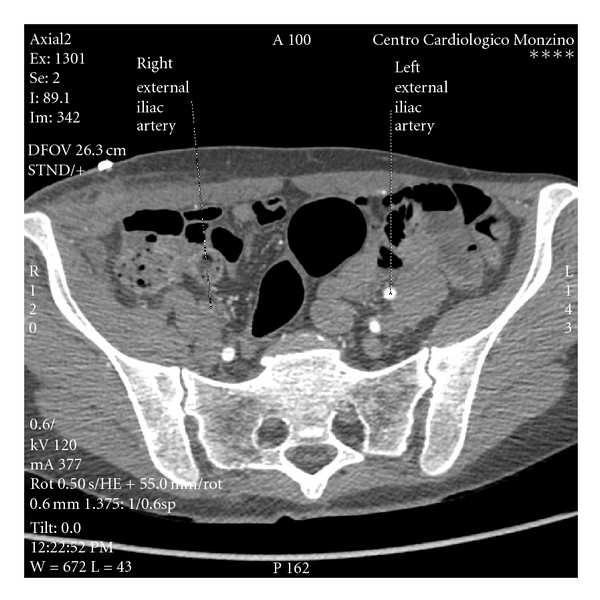
Axial view of right external iliac artery thrombosis. No contrast-enhancement is visible.

A 48-year-old woman was admitted to our hospital with diagnosis of subacute thrombosis of the right external iliac and common femoral artery, following HILP for recurrence of right limb melanoma. HILP was performed 3 weeks prior: common right femoral artery and vein were isolated with open technique, heparin was given, and then the vessels were cannulated and connected to a perfusion circuit providing membrane oxygenation and a heat exchanger (perfusate temperature 38.5–40°C). Melphalan was given in a dose of 10 mg/L of tissue volume and human recombinant TNF-*α* in a dose of 2 mg. A duplex ultrasound of the right iliac and femoral vessels performed preoperatively to assess vessel size documented good quality vessels and, of note, the absence of peripheral vascular disease (a contraindication to HILP). Several days after, another duplex scan was obtained, as postprocedural routine, to document regular vessels patency but, unexpectedly, it showed a postocclusive demodulated duplex signal, with the femoro-popliteal-tibial axis reperfused by collateral circulation, being the patient asymptomatic for claudicatio or limb pain and with a slight clinical evidence of limb ischemia. We carried out a multidetector 64-slice CT angiography (collimation 64 mm × 0.625 mm; tube current 300 mA, tube voltage 120 Kv) after injection of two intravenous bolus of contrast dye (Iomeron 350 mg/mL; first bolus 60 mL volume, 4 mL/s flow; second bolus 40 mL volume, 2 mL/sec flow). The evaluation was completed by volume rendering and multiplanar reconstructions (MPR). As shown in Figure 1, the external iliac artery was completely occluded by a thrombus and reperfusion flow was appreciated in the distal common femoral artery and in profunda femoris by collateral circulation. The patient underwent surgical intervention consisting in iliac artery-femoral artery bypass with a Dacron vascular graft 8 mm in caliper. Postprocedural hospital stay was uneventful, and she was discharged home on day 11. 

## 3. Discussion

**Figure 2:**
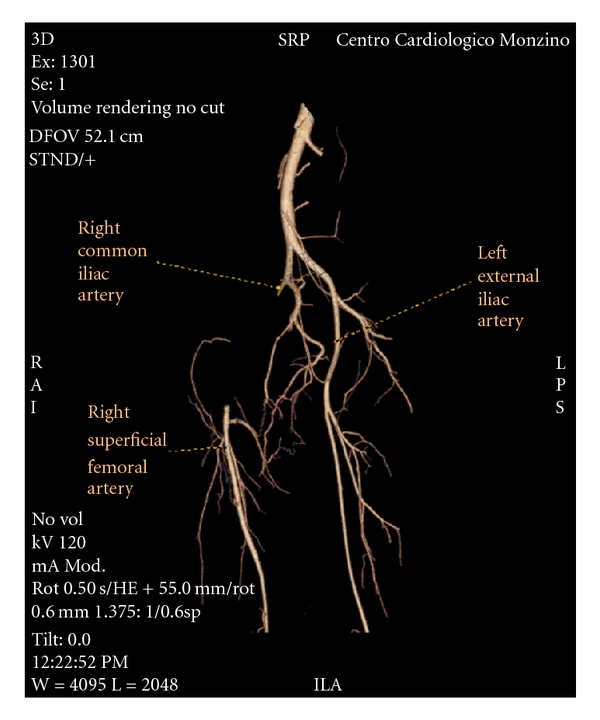
Postprocessing 3D reconstruction of the iliac stenosis and the collateral circulation reperfusing mainly the profunda femoris from the omolateral internal iliac artery.

 Despite promising data, HILP is only used in a few centres worldwide for the principal reason that although the procedure is simple in concept, it is surgically and technically complex, and demanding in practice [3]. Acute/subacute arterial occlusion is one of the most feared vascular complications of HILP, located nearly always in the external iliac-femoral artery axis (Table 1). Usually patients who experience such condition are females. Regarding the cause of thrombosis and the role of the drugs used, there is evidence that TNF-*α* targets the tumor vascular endothelial cells, increases tumor vascular leakiness, decreases tumor blood flow, and selectively disrupts tumor endothelial junctions by changing localization of VE-Cadherin. For this reason, endothelial damage cannot be excluded as a possible cause that may amplify surgical trauma, leading to subacute thrombosis. In the absence of peripheral vascular disease, subacute external iliac-femoral artery occlusion rarely produces limb-threatening symptoms, especially in patients who often are forcedly bed-bound. In such a localized obstruction, the potential for collateral circulation is great. The major visceral routes for collateral pathways are the lumbar and internal iliac to profunda femoris (Figure 2). Multidetector CT angiography with MPR is now considered the noninvasive tool of choice for imaging the vascular system and is appropriate for preprocedural planning and postprocedural control, in particular for lower extremity inflow and runoff studies [4]. In case of HILP-related vascular complications, its unvaluable aid helps surgeons to plan and to offer the best treatment option. In conclusion, major vascular complications following HILP may develop in the early postoperative phase; close post-HILP monitoring of the peripheral circulation in perfused limbs is necessary in early detection of these complications, as early surgical reintervention may lead to a good outcome with no sequelae. 

## Figures and Tables

**Table 1:** Incidence of vascular complications after HILP in the literature.

Reference	Arterial thrombosis	Thrombosis site	HILP drugs
Rochlin (1965)	2/200 (1%)	Femoral artery	Melphalan
Fontaine (1974)	1/56 (1.8%)	External iliac artery	Melphalan
Bulman (1980)	4/75 (5.3%)	Femoral artery	Melphalan
Krementz (1994)	6/703 (0.9%)	Femoral artery	Drug combinations
Hohenberger (1994)	3/342 (0.9%)	External iliac artery	Drug Combinations
Klicks (1998)	10/466 (2.1%)	External iliac artery	TNF-*α* + Melphalan
Rossi (2004)	1/20 (5%)	Femoral artery	TNF-*α* + Melphalan
Grunhagen (2005)	1/217 (0.5%)	Mesenteric artery	TNF-*α* + Melphalan
